# ICTV Virus Taxonomy Profile: *Fimoviridae*

**DOI:** 10.1099/jgv.0.001143

**Published:** 2018-09-11

**Authors:** Toufic Elbeaino, Michele Digiaro, Nicole Mielke-Ehret, Hans-Peter Muehlbach, Giovanni P. Martelli, ICTV Report Consortium

**Affiliations:** ^1^​ CIHEAM-IAMB, Via Ceglie 9, 70010 Valenzano, Bari, Italy; ^2^​ Biocentre Klein Flottbek, University of Hamburg, Ohnhorststrasse 18, Hamburg 22609, Germany; ^3^​ Università Aldo Moro, Bari, Via G. Amendola 165/a, 70126 Bari, Italy

**Keywords:** *Fimoviridae*, *Emaravirus*, taxonomy, ICTV Report

## Abstract

Members of the family *Fimoviridae,* order *Bunyavirales* are plant viruses with segmented, linear, single-stranded, negative-sense RNA genomes. They are distantly related to orthotospoviruses and orthobunyaviruses of the families *Tospoviridae* and *Peribunyaviridae*, respectively. The family *Fimoviridae* includes the genus *Emaravirus*, which comprises several species with *European mountain ash ringspot-associated emaravirus* as the type species. Fimoviruses are transmitted to plants by eriophyid mite vectors and induce similar characteristic cytopathologies in their host plants, including the presence of double membrane-bound bodies in the cytoplasm of the virus-infected cells. This is a summary of the International Committee on Taxonomy of Viruses (ICTV) Report on the taxonomy of the *Fimoviridae*, which is available at www.ictv.global/report/fimoviridae.

## Virion

**Fig. 1.**
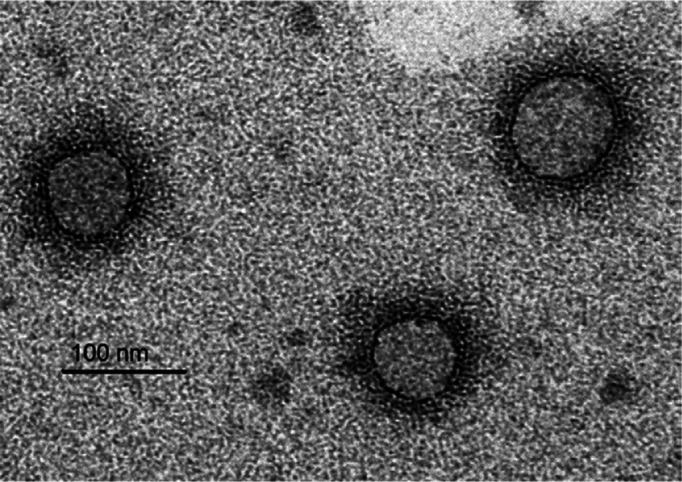
Immunosorbent electron micrograph of virions of European mountain ash ringspot-associated virus (Courtesy Inga Ludenberg, University of Hamburg, Germany).

Fimoviruses have enveloped, approximately spherical, virions, with a diameter of 80–100 nm (Table 1, Fig. 1).

**Table 1.** Characteristics of the family *Fimoviridae*

Typical member:	European mountain ash ringspot-associated virus, Hamburg (RNA1: AY563040; RNA2: AY563041; RNA3: DQ831831; RNA4: DQ831828), species *European mountain ash ringspot-associated emaravirus*, genus *Emaravirus,* family *Fimoviridae,* order *Bunyavirales*
Virion	Approximately spherical and enveloped with a diameter of 80–100 nm
Genome	Four to eight segments of negative-sense ssRNA (12.3–18.5 kb in total)
Replication	No information
Translation	From capped mRNAs (produced by ‘cap snatching’ from host mRNAs) which are complementary to the vRNAs
Host range	Cercis, fig, kiwi, maize, pigeonpea, raspberry, rose, Sorbus, wheat
Taxonomy	Single genus including nine species

## Genome

**Fig. 2.**
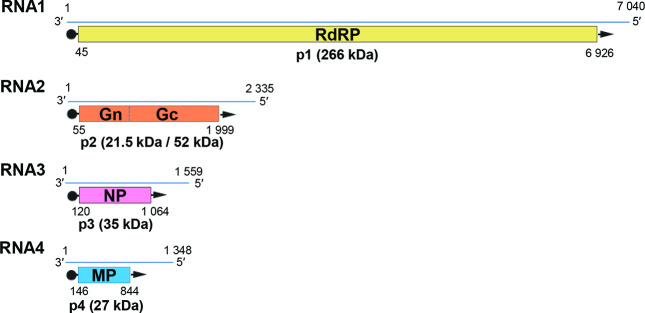
Coding strategy and genome segments of European mountain ash ringspot-associated virus. Arrows represent the virion-complementary sense RNA, from which the proteins shown are translated. RdRP, RNA-dependent RNA polymerase; Gn and Gc, putative glycoproteins cleaved (dotted line) from the precursor molecule; NP, nucleocapsid protein; MP, putative movement protein.

The virus genome comprises four (European mountain ash ringspot-associated virus) to eight (High Plains wheat mosaic virus) segments of negative-sense ssRNA comprising 12.3 to 18.5 kb in total [1, 2] (Fig. 2). Genomic RNAs are not capped or polyadenylated and all contain complementary sequences (18–20 nt, depending on the RNA segment) at their 5′- and 3′-termini [3, 4].

## Replication

The complementary strand of each genome RNA segment encodes a single protein. In order from RNA1 to RNA4, the encoded proteins are: the RNA-dependent RNA polymerase (p1, 264–269 kDa, 226.9 kDa in Actinidia chlorotic ringspot-associated virus), a glycoprotein precursor (p2, 73–76.6 kDa) that is predicted to be cleaved into products of 21.5–25.0 and 51–52 kDa, the nucleocapsid protein (p3, 32–35.6 kDa) and a putative movement protein (p4, ca. 40.5–43.6 kDa). The functions of the proteins encoded by RNAs 5–8 remain unknown.

Like other negative-sense ssRNA multipartite viruses, some fimoviruses are known to use ‘cap snatching’ to initiate transcription and facilitate translation of their mRNAs [5, 6].

## Taxonomy

Viruses of the family *Fimoviridae* (order *Bunyavirales*) are related to viruses in the families *Tospoviridae* and *Peribunyaviridae* in that they share: (i) a multipartite, negative-sense, single-stranded RNA genome of four to eight segments; (ii) high sequence identity with orthologous proteins of members of the order *Bunyavirales* at equivalent genome positions in the first three RNAs (corresponding to L, M and S RNA segments); (iii) five conserved motifs (A–E) in the amino acid sequence of their RNA-dependent RNA polymerase, similar to those in the L segment-encoded protein of members of the order *Bunyavirales*; (iv) enveloped virions; (vi) stretches of nucleotides at both the 5′- and 3′-termini of all RNA segments that are nearly complementary to each other. These terminal sequences are conserved in all of the genomic RNAs of fimoviruses, and are similar, but not identical, to those of other members of the order *Bunyavirales.*

Phylogenetic trees constructed with fimovirus RNA-dependent RNA polymerase, glycoprotein precursor and nucleocapsid protein sequences display two main clusters [7, 8].

## Resources

Full ICTV Online (10th) Report: www.ictv.global/report/fimoviridae.
